# Reproduction and Wing Differentiation of Gynoparae Are Regulated by Juvenile Hormone Signaling in *Aphis gossypii*

**DOI:** 10.3390/insects16060559

**Published:** 2025-05-25

**Authors:** Liuyu Wang, Jingli Lv, Xiangzhen Zhu, Kaixin Zhang, Qingyu Shi, Li Wang, Weihua Ma, Jichao Ji, Junyu Luo, Jinjie Cui

**Affiliations:** 1 State Key Laboratory of Cotton Bio-Breeding and Integrated Utilization, Institute of Cotton Research, Chinese Academy of Agricultural Sciences, Anyang 455000, China; liuyuwang2021@163.com (L.W.); lv1519361607@163.com (J.L.); zhuxiangzhen318@163.com (X.Z.); zhangkaixin@caas.cn (K.Z.); shiqy20159@126.com (Q.S.); wangli08zb@126.com (L.W.); luojunyu1818@126.com (J.L.); aycuijinjie@163.com (J.C.); 2 Hubei Insect Resources Utilization and Sustainable Pest Management Key Laboratory, College of Plant Science and Technology, Huazhong Agricultural University, Wuhan 430070, China; 3 State Key Laboratory of Cotton Bio-breeding and Integrated Utilization, School of Agricultural Sciences, Zhengzhou University, Zhengzhou 450001, China; 4 Western Agricultural Research Center, Chinese Academy of Agricultural Sciences, Changji 831100, China

**Keywords:** *Aphis gossypii*, gynoparae, temporal transcriptome analysis, juvenile hormone, wing development, reproduction

## Abstract

Gynopara is a specific winged type in the life history of *Aphis gossypii*. As a key reproduction mode between parthenogenesis and sexual reproduction, it lays sexual females in late autumn. However, little is known about gynoparae because of its rare presence in the wild and the lack of its detailed descriptions. In this study, we investigated the morphological characteristics, ovary maturation process, and key signaling pathways during the development of gynoparae of *A. gossypii*. With the extension of development stage, the gynoparae exhibited gradually enlarged and thickened abdomen, deepened color. Obviously differentiated wing primordia in the second instar nymphs. Two nested U-shaped zones containing a series of waxy secreta spots were present on the abdomen of the fourth instar nymphs, and these morphological characteristics could be used as important markers for identifying gynoparae. Temporal transcriptomic analysis suggested that juvenile hormone (JH) might be involved in regulating the development of gynoparae. After the application JH analog kinoprene to the first instar nymphs, almost all treated individuals failed to complete wing differentiation, and most lost the ability to produce progeny, suggesting the destructive effect of JH on wing differentiation and reproduction of gynoparae. Moreover, exogenous addition of kinoprene also significantly altered the expression levels of four key genes responsible for the synthesis and degradation of JH. Our findings reveal the mechanism by which JH regulated wing differentiation and reproductive capacity of gynoparae, which lay a foundation for the further research on reproduction mode switch in aphids in the future.

## Simple Summary

Gynoparae, an exclusively winged morph contributing to the reproductive mode transition from parthenogenesis to gamogenesis of *Aphis gossypii*, were studied to characterize their developmental features. Typical morphological characteristics of gynoparae included gradual abdominal enlargement, darkened coloration, distinct wing primordia in second instar nymphs, and two nested U-shaped abdominal zones with wax-secreting spots in fourth instar nymphs. Transcriptomic analysis indicated juvenile hormone (JH) signaling involving in regulating the development of gynoparae. Application of the JH analog kinoprene to first instar nymphs of gynoparae disrupted their wing differentiation, abolished the reproductive capacity, and significantly altered the expression level of JH synthesis and degradation-related genes. These findings reveal JH-mediated molecular signaling governing wing development and reproduction in gynoparae, and provide new insights into the regulation mechanism about wing differentiation and reproductive ability of aphids.

## 1. Introduction

*Aphis gossypii* Glover (Hemiptera: Aphididae), also well-known as cotton or melon aphid, is a polyphagous agricultural pest distributed worldwide [1]. It can directly suck phloem sap from hundreds of different host plants, spread various plant viruses, and disrupt plant photosynthesis by secreting honeydew, thus causing huge economic losses to agricultural production [2,3,4]. In temperate regions, most of the life cycle of the cotton aphid is the heteroecious holocyclic type. These types of aphids such as *A. fabae*, *A. glycines*, and *Myzus persicae* typically undergo cyclical parthenogenesis. Their entire life cycle comprises one sexual reproduction and multiple asexual reproductions [5], and usually involves multiple host migrations [6,7]. The heteroecious holocyclic type of aphid accounts for more than 90% of all aphid species, and this type tends to reproduce offspring in parthenogenesis during the spring and summer. In late autumn, short daylight conditions induce the production of gynoparae, which migrate to the winter host plants and lay sexual females. Then, the sexual females mate with the males to produce overwintering eggs, eventually completing their whole life cycle [8].

**Figure 1:**
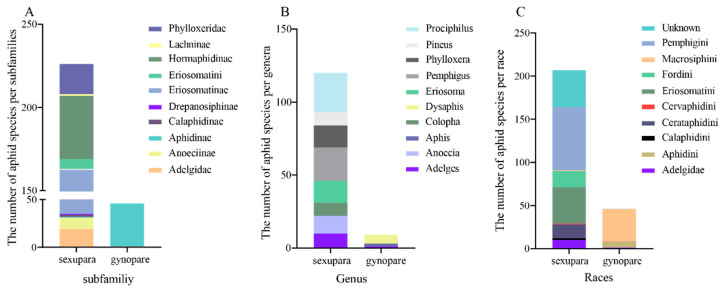
Distribution of gynoparae and sexuparae in Aphididae at the level of subfamily (**A**), genus (**B**), and race (**C**). The top 10 categories with the largest number of individuals at these three taxonomic levels are presented, and the rest are exhibited in Supplementary Table S2.

As specific winged aphids, gynoparae belonging to heteroecious holocyclic aphids can receive signals from the winter host plant [9,10], which prompts them to migrate to the winter host plant and produce sexual females to enter the sexual reproduction phase [11,12,13]. After the completion of host transfer and dispersal, the gynoparae stage is a crucial reproductive transition period from asexual reproduction to sexual reproduction for heteroecious holocyclic aphids. Gynoparae, as a promising model, are conducive to revealing reproductive mode switch and wing pattern differentiation in heteroecious holocyclic aphid species, but few reports on it are available. So far, only 46 species of aphids have been reported to have gynoparae (Figure 1).

Reproduction is one of the most fundamental behavioral activities of insects and an essential means to ensure the sustainability and prosperity of insect populations [14]. Juvenile hormone (JH) is synthesized by the corpora allata (CA) and secreted into the hemolymph, and it is widely acknowledged as one of the most vital regulatory factors in insect reproduction [15]. JH has been reported to regulate the formation of vitellogenin, which is a prerequisite for oviposition and subsequent embryonic development in most insects [16]. For example, in *Drosophila melanogaster*, the JH signal can activate the production of vitellogenin in the fat body, promote ovulation, and maintain the egg shape [17]. In *Rhodnius prolixus*, a complex endocrine signaling network regulates the reproductive cycle, namely insulin and allatoregulatory neuropeptides, and drives an increase in JH content, while in return, JH can control its own biosynthesis through negative feedback regulation [18]. In *Sogatella furcifera*, insulin and the AA/TOR pathway can regulate reproduction by controlling anabolic processes and the secretion of JH [19,20,21]. In *Acyrthosiphon pisum*, the application of 100 ng JH increases adult weight and fecundity, indicating that JH can regulate the body weight and reproductive response [22]. The exploration of the reproductive regulation mechanism of aphids is beneficial to developing sustainable aphid control strategies. Although the treatment of gynoparae of *A. fabae*, *M. persicae*, and *Megoura viciae* with JH or its analog kinoprene has been reported to change their reproductive modes, whether or how JH can regulate the reproduction of gynoparae of the cotton aphid remains largely unknown [23,24].

JH can regulate insect wing dimorphism and wing development. A widely accepted hypothesis is that at a certain critical development period, an increase in the JH titer completely or partially blocks the normal morphogenesis of wings, thus leading to the production of a short-winged or wingless morphology [25]. Dingle et al. [26] have found that JH-mediated genetic and environmental changes can influence wing polymorphisms in species of the soapberry bug (Jadera hematoma), evolving rapidly in new host plants. McCaffery et al. [27] have found that the absence of JH in the last nymphal instar results in the formation of long-winged adults, whereas the addition of an exogenous JH analogue leads to the formation of short-winged adults, indicating that exogenous JH affects the wing length in Zonocerus variegatus. Ishikawa et al. [28] treated the third instar winged *Megoura crassicauda* nymphs with JHIII, resulting in the generation of winged/wingless intermediates and the increased ecdysis of juvenile individuals. These findings jointly suggest that JH plays an important role in the differentiation of wing morphology during post-embryonic development. In *Eumeta variegata*, the application of a high concentration of JH analogue (methoprene, 5 μg/mL) and 20E (20-hydroxyecdysone, 1 μg/mL) induces apoptosis in the male wing [29]. Additionally, the interplay between endocrine and cellular signaling pathways can also affect the plasticity of insect wings, and several pathways including juvenile hormone/ecdysteroid hormones, insulin signaling, and JNK signaling have been reported to regulate the wing polymorphism collectively in multiple insects such as crickets and *Bombyx mori* [30,31].

In *A. fabae*, gynoparae treated with kinoprene exhibit abnormal development, with deformed wing and mixed ovaries [24], indicating that JH can regulate both wing differentiation and reproduction. However, Hardie et al. have found that the addition of high-dose JH to the gynoparae of *A. fabae* affects only reproduction but not wing differentiation, as indicated by the fact that alate gynoparae adults induced by JH fail to reproduce, but they can fly [32]. These inconsistent reports suggest that the roles of JH in regulating reproduction and wing formation are complicated, which remains to be further investigated.

In this study, we first statistically analyzed the proportion of aphid species in which gynoparae were reported, as well as their distribution in different taxa, and systematically investigated the morphological characteristics and developmental dynamics of gynoparae of *A. gossypii*. Through temporal transcriptome analysis, we screened key genes and signaling pathways related to the JH synthesis, wing differentiation, and reproduction in gynoparae. We further explored the regulatory effects of JH on wing differentiation and reproductive development in gynoparae by treating the gynoparae with kinoprene (JH analog). Our research provides new perspectives for further exploration of the regulatory mechanism underlying wing dimorphism and reproductive mode switch in gynoparae of aphids.

## 2. Methods and Materials

### 2.1. Gynoparae in Aphids

Two types of individuals (sexuparae and gynoparae) in aphid species can produce sexual females. The former can generate sexual females, males, and parthenogenetic females, whereas the latter only produce exclusively sexual females [33]. To date, the distribution of sexuparae and gynoparae in aphid species remains unclear. In this study, we surveyed sexuparae and gynoparae distribution, and the retrieval results are listed and categorized at different taxonomic levels (Table S2).

### 2.2. Gynoparae Induction

*A. gossypii*, collected originally in Anyang (Henan, China), was reared on cotton seedlings under controlled laboratory conditions for more than 50 generations before all the experiments (25 ± 1 °C, 75% relative humidity, and 16 h light:8 h dark photoperiod). Gynoparae were induced by rearing newly born wingless parthenogenetic nymphs under short daylight (SD) conditions (18 ± 1 °C, 75% relative humidity, and 8 h light:16 h dark photoperiod) [34]. Especially, newly born viviparous nymphs reared under SD conditions exclusively produced gynoparae within their first reproductive cycle (approx. one week) in adulthood after eclosion, and no winged and wingless virginoparae were produced in this standardized induction protocol. Gynoparae at different development stages (first to fourth instar nymphs and adults) were identified through corresponding development time and molting times [35,36,37].

### 2.3. Morphological Characteristics and Fertility

The newly born gynoparae (within 12 h post-birth) were reared on cotton leaves in petri dishes containing 1.8% agar (*m*/*v*). The molting of gynoparae was observed and recorded every 24 h throughout its development process. Biological data such as body length and wing length of adult aphids at each developmental stage were measured using a SteREO Discovery V8 microscope (Zeiss, Oberkochen, Germany). After statistical data analysis, GraphPad Prism 8.0.1 was used for plotting. When gynoparae entered the adult stage, the number of their daily produced offspring was observed and recorded. The first–fourth instar nymphs and adult gynoparae were dissected to explore their ovarian development and embryogenesis throughout their development.

### 2.4. Preparation of RNA Sequencing Samples

Based on morphological characteristics, a total of 15 samples were collected from the gynoparae across all five developmental stages (first–fourth instars and adult), with three biological replicates per stage. Each sample contained at least 30 aphids. All samples were frozen immediately in liquid nitrogen and stored at −80 °C. Total RNA of each sample was extracted with RNAiso Plus kit (Takara, Beijing, China) according to the operation instruction, respectively. After the sample test, the corresponding cDNA library was constructed and sequenced on Illumina NovaSeq6000 platform, respectively. The raw data were subjected to quality control to remove the adapters and low-quality reads, and finally, clean reads were obtained (Table S4: Summary of RNA-Seq data).

### 2.5. Transcriptome Assembly and Gene Annotation

The obtained clean reads were aligned against the published genome of *A. gossypii* (version ASM2018417v2) using HISAT2 [38,39]. Then, the aligned reads were assembled using String Tie, and the transcriptome was reconstructed for subsequent analysis [40]. The new genes were aligned against the NR, Swiss-Prot, COG, KOG, and KEGG databases, and their functional annotation was performed using the Inter Pro integrated database. The predicted amino acid sequences of new genes were aligned against the Pfam database using HMMER software v3.4 to obtain the annotation information [41].

### 2.6. Identification of Differentially Expressed Genes

DESeq2 software (v1.48.1) was used for identifying the differentially expressed genes (DEGs) across the development of gynoparae, with the thresholds of |log_2_ FC (fold change)|≥ 2 and false discovery rate (FDR) < 0.05 [42]. The KEGG (Kyoto Encyclopedia of Genes and Genomes) pathway enrichment analysis was performed in order to analyze the biological functions of DEGs and identify the pathways related to the wing differentiation and reproduction of gynoparae of the cotton aphid, with the criterion of Q-value < 0.05.

### 2.7. Validation of RNA-Seq Data by Real-Time Quantitative Polymerase Chain Reaction (RT-qPCR)

To verify the reliability of the RNA-Seq results, 12 DEGs were randomly selected for RT-qPCR. The primers were designed using Primer Premier 6.0, and their sequences are listed in Table S1. RT-qPCR was performed on a Light Cycler 480 machine (Roche Diagnostics, Risch-Rotkreuz, Switzerland) with a 20 μL reaction system containing 2 μL cDNA template, 7.2 μL nuclease-free water, 0.4 μL forward primer, 0.4 μL reverse primer, and 10 μL 2 × TransStart^®^ Top Green qPCR SuperMix (+DyeI/+DyeII) (TransGen Biotech, Beijing, China, AQ131). The RT-qPCR program was as follows: 94 °C for 30 s, 40 cycles of 94 °C for 5 s, 55 °C for 1 s, and 72 °C for 10 s. The relative expression levels of genes were calculated using the 2^−ΔΔCT^ method and normalized with the housekeeping gene *GAPDH* as an internal control [43,44].

### 2.8. Effects of JH Analogue Kinoprene on Gynoparae

The first instar gynoparae nymphs were treated with kinoprene (JH analogue) (Cat: DR-CA14538000) to explore effects of the JH-signaling pathway on the wing differentiation and reproduction of gynoparae. Each treatment included four biological replicates, with each replicate consisting of 25 gynoparae nymphs. Initially, 0.1% and 0.01% kinoprene solutions were prepared, respectively. Specifically, 1.0 mL of 1% kinoprene stock solution (in acetone) was emulsified with 9.0 mL of 0.1% Tween-20 solution (in water) with shaking in a vial to obtain 0.1% kinoprene solution. The resulting 0.1% kinoprene solution was diluted by 0.1% Tween-20 solution to prepare 0.01% kinoprene [23,24]. The 0.1% and 0.01% kinoprene were sprayed onto the leaves with a 5 mL glass spray bottle, respectively. After the leaves were dried, the first instar gynoparae were transferred to the leaves, and kinoprene-sprayed leaves were replaced every 2 days. The gynoparae were sampled at 2 d, 4 d, 6 d, and 8 d post-rearing, and the relevant phenotypes were investigated. The morphological characteristics and dynamics of the nymphs were observed until they emerged into adults. The phenotypes of adult insects were classified according to offspring number and wing deformation degree. One-way ANOVA analysis was performed to reveal the significant differences between the experimental groups; *p* < 0.05 was considered statistically significant.

To examine the response of JH pathway in gynoparae to kinoprene, the expression levels of 4 key JH biosynthesis-related genes were detected by RT-qPCR, including *JHE* (juvenile hormone esterase), *JHEH* (juvenile hormone epoxide hydrolase), *JHDK* (juvenile hormone diol kinase), and *JHAMT* (juvenile hormone acid methyltransferase). In one experiment, the samples were collected at 2 d after 0.1% kinoprene treatment of the first instar gynoparae for the determination of the relative expression levels of the above-mentioned 4 genes. In another experiment, after 2 d treatment with 0.1% kinoprene, the samples were transferred to unsprayed leaves and collected at 6 d post-transfer for the determination of gene relative expression levels.

## 3. Results

### 3.1. Distribution of Gynoparae in Aphididae

In this study, we surveyed the distribution of sexuparae and gynoparae in Aphididae and found that sexuparae were obtained in 229 races, belonging to 13 subfamilies, 62 genera, and 11 races, respectively (Figure 1, Table S2). At the subfamily level, sexuparae were recorded mainly in Eriosomatinae (128), followed by Hormaphidinae (38) (Figure 1A). At the genera level, sexuparae were mostly found in *Prociphilus* (27), followed by genera *Pemphigus* (23) (Figure 1B). At the race level, sexuparae were observed primarily in Pemphigini (73) and Eriosomatini (42) (Figure 1C). In contrast, gynoparae were obtained in 46 species, belonging to 2 subfamilies, 25 genera, and 3 races, respectively. At the subfamily level, gynoparae were recorded mainly in Aphidinae (45) and *Adelgidae* (1) (Figure 1A). At the genera level, gynoparae were mostly found in Tuberocephalus (7) (Figure 1B). At the level of race, gynoparae were observed primarily in Macrosiphini (38) (Figure 1C).

### 3.2. Morphology and Embryogenesis of Gynoparae

**Figure 2:**
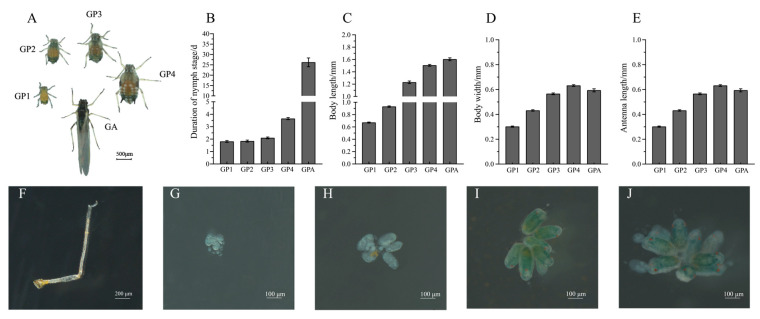
**Morphology, ovarian development, and embryogenesis of cotton aphid gynoparae.** (**A**) Morphological dynamics of gynoparae during five developmental stages. (**B**) Duration time of five developmental stages of gynoparae. (**C**–**E**) Body length, body width, and antenna length of gynoparae at five developmental stages. (**F**) Hind leg of gynoparae. (**G**–**J**) Ovarian morphology of gynoparae at second–fourth instar nymph and adult stages.

Wing differentiation is one of the most pronounced external morphological changes during the development of gynoparae. The wing differentiation of gynoparae of *A*. *gossypii* fell into four stages: wing primordia differentiation (the first instar), wing sac formation (the second and third instar), wing bud formation (the fourth instar), and wing eclosion stage (adult). However, the wings of first instar gynoparae were at the stage of wing primordia differentiation, and morphological changes in wing formation could not be observed from external morphology (Figure 2A). Apparent wing formation could be observed with the naked eye from the second instar nymphs to the adults of gynoparae. The thorax of the second instar gynoparae was slightly protruded, and the body wall was thickened on both sides of the thorax and abdomen. Additionally, the thorax of the third instar gynoparae was obviously protruded. Further, wing buds of fourth instar gynoparae were clearly visible and occupied the whole thorax, and the black wing apexes reached the second and third segments of the abdomen. Finally, in the adult stage, the wings of gynoparae were fully extended after eclosion (Figure 2A).

Additionally, the duration of development of the first–fourth instar nymphs of gynoparae was 1.81 d, 1.84 d, 2.10 d, and 3.65 d, respectively, and the longevity of gynoparae adults was 28.23 d (Figure 2B). The body size was gradually increased with development, of which the mean body length at each development stage (first–fourth instar nymphs and adults) was 0.67 mm, 0.94 mm, 1.18 mm, 1.51 mm, and 1.60 mm, respectively, and the mean body width at each development stage was 0.30 mm, 0.43 mm, 0.55 mm, 0.63 mm, and 0.59 mm, respectively (Figure 2C,D). In addition, the mean antennal length of gynoparae throughout the development period gradually increased from the first instar nymph to the adult, which was 0.30 mm, 0.45 mm, 0.62 mm, 0.91 mm, and 1.00 mm, respectively (Figure 2E). The wing length of the adult gynoparae was 2.90 mm (Table S3).

Significant changes in body color and shape were also observed during the development of the gynoparae. The body of the first instar gynoparae was brown with a black head and cornicles. The second to third instar gynoparae had more black areas on the head, thorax, and cornicles, and the body color of the fourth instar gynoparae became darker, with two nested U-shaped zones containing a series of waxy secreta spots on the brownish abdomen (Figure 2A). The epidermis of the gynoparae was completely blackened and hardened at adulthood, and the distal end of the hind foot tibial segment was black (Figure 2F). The dissection results exhibited that developing early embryos existed in the ovaries of the second and third instar gynoparae, and greenish embryos with red eyes were visible in the fourth instar of gynoparae, but eventually only the first embryo in each ovary matured into an adult (Figure 2G–J). The average number of offspring produced by each gynoparae was 7.32 (Table S3).

### 3.3. Transcriptome Data and Their Reliability Analysis

**Figure 3:**
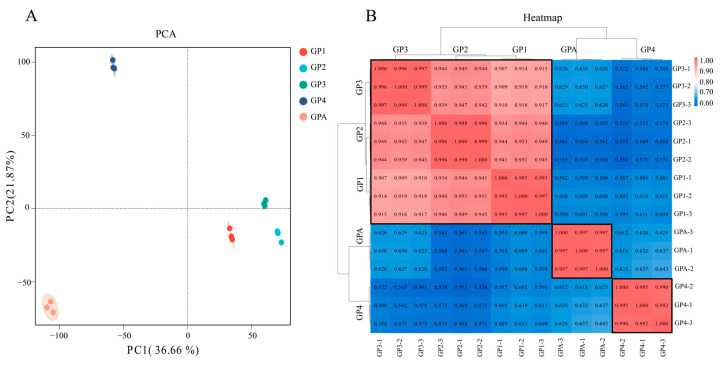
Principal component analysis (PCA). (**A**) and Pearson correlation analysis heatmap (**B**) among all samples from 5 development stages. Samples were clustered into 5 groups, namely, GP1, GP2, GP3, GP4, and GPA, which indicate the first, second, third, and fourth instar nymphs and adults of gynoparae, respectively. GP1-1, GP1-2, and GP1-3 represent three biological replicates of GP1, respectively, and so on. In the correlation heatmap, dark red represents strong correlation, and dark blue denotes a weak correlation. Each column and row correspond to the correlation between one sample and other 14 samples (including itself).

Ultimately, 95.55 Gb clean reads were obtained from all gynoparae samples, with an average Q30 score of 85.46%, and 92.92% of clean reads were aligned against a reference genome (ASM2018417v2) (Table S3). Principal component analysis (PCA) showed that samples at different development stages exhibited obvious separation, but the samples from different replicates at the same development stage were close to each other (Figure 3A). The cluster analysis indicated that gynoparae samples fell into three categories, and the colors of GP1, GP2, and GP3 (gynoparae at first, second, third instar stage) were close, but they were obviously different from those of GP4 and GPA (gynoparae at fourth instar stage and gynoparae at adulthood), indicating that GP1, GP2, and GP3 were highly correlated, which was in accordance with the PCA results (Figure 3B).

Subsequently, the expression patterns of 12 randomly selected genes during the gynoparae development obtained from qPCR were consistent with the RNA-Seq results, confirming the reliability of RNA-Seq (Figure S1). Therefore, the RNA-Seq data were qualified for subsequent analysis.

### 3.4. KEGG Enrichment Analysis of DEGs

**Figure 4:**
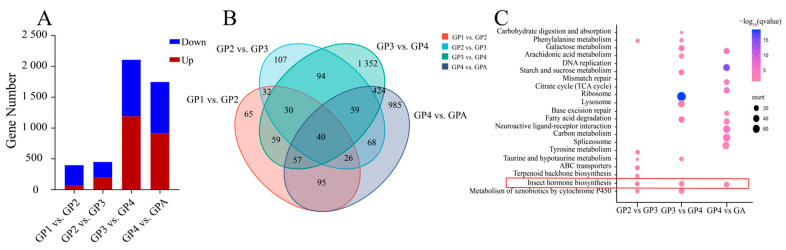
(**A**) Number of DEGs identified in the pairwise comparisons of GP1 vs. GP2, GP2 vs. GP3, GP3 vs. GP4, and GP4 vs. GPA. (**B**) Venn diagram of DEGs in the pairwise comparison of GP1 vs. GP2, GP2 vs. GP3, GP3 vs. GP4, and GP4 vs. GPA. GP1, GP2, GP3, GP4, and GPA represent the first, second, third, and fourth instar nymphs and adults of gynoparae, respectively. (**C**) KEGG pathway enrichment analysis of differentially expressed genes (DEGs) across the development of gynoparae. The proportion of genes enriched in the target pathway indicates enrichment degree. The color of the bubble from red to blue indicates gradually decreased *p*-value. The size of the bubble indicates the number of DEGs enriched in the pathway; the larger the bubble, the more genes enriched.

KEGG enrichment was performed on differentially expressed genes (DEGs) in the comparison of gynoparae development stages (Figure 4A). No signaling pathways were enriched with the 404 DEGs in the first vs. second instar gynoparae (GP1 vs. GP2). In the comparison of the second instar gynoparae (GP2) vs. the third instar gynoparae (GP3), 456 DEGs were identified and mainly enriched in signaling pathways related to insect hormone biosynthesis, amino acid metabolism, and terpenoid backbone biosynthesis. A total of 2115 DEGs were identified in GP3 vs. GP4 and mainly enriched in signaling pathways related to insect hormone biosynthesis, carbohydrate metabolism (such as starch and sucrose metabolism and galactose metabolism), neuroactive ligand–receptor interaction, and fatty acid degradation. In the comparison of GP4 vs. GPA (gynoparae adult), 1754 DEGs were identified and mainly enriched in signaling pathways related to genetic information processing (such as DNA replication and base excision repair), carbohydrate metabolism (such as citrate cycle and carbon metabolism), insect hormone biosynthesis, and fatty acid degradation (Figure 4B,C).

Notably, the DEGs from the three comparisons (GP2 vs. GP3, GP3 vs. GP4, and GP4 vs. GA) were all significantly enriched in signaling pathways of insect hormone biosynthesis (IHB) (Figure 4C, Table S5). Specifically, compared to the second instar nymphs, four and five IHB-related genes in the third instar nymphs were significantly up-regulated and down-regulated, respectively. Compared to the third instar nymphs, ten and six IHB-related genes in the fourth instar nymphs were significantly up-regulated and down-regulated, respectively. Compared to the fourth instar nymphs, ten and four IHB-related genes in gynoparae adults were significantly up-regulated and down-regulated, respectively. The morphology and embryogenesis dynamics of gynoparae as well as the expression level changes of IHB-related genes across the gynoparae development collectively suggested that insect hormones (especially JH) might be involved in regulating the development of gynoparae, especially in their wing differentiation and reproduction.

### 3.5. Effects of Kinoprene on Wing Differentiation and Reproduction of Gynoparae

**Figure 5:**
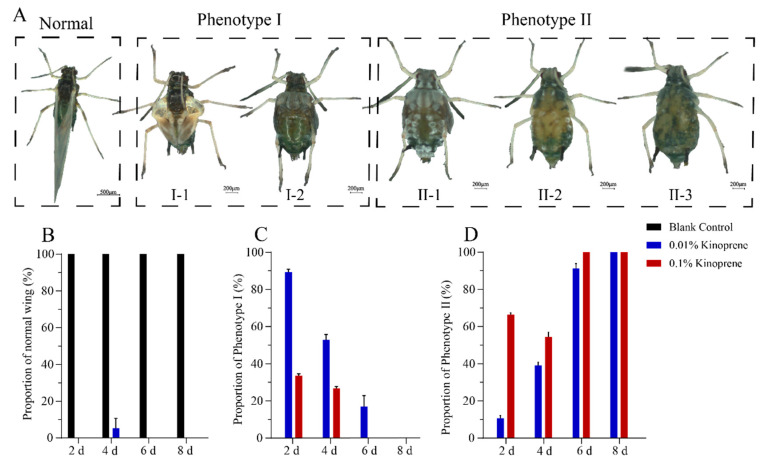
Phenotypes of gynoparae exposed to kinoprene at two concentrations (0.1% and 0.01%). (**A**) The two representative deformed phenotypes. Normal phenotype indicates normal wings and reproduction of gynoparae. Deformity I refers to 4 times of molting with deformed wing (I-1) or unfolded (I-2) wing but with ability to produce offspring. Deformity II denotes under-developed wing (II-1), partial degenerated wing (II-2), or fully degenerated wing (II-3) wing and inability to produce offspring. (**B**–**D**) The proportion of normal wing, Phenotype I and Phenotype II corresponding to kinoprene-treated (0.1% and 0.01%) gynoparae after 2 d, 4 d, 6 d, and 8 d.

Kinoprene was applied to the first instar gynoparae nymphs to explore the regulatory effect of JH on the wing differentiation and reproduction of gynoparae. The results showed that 20–85% of GP1 exposed to 0.1% and 0.01% kinoprene for 2 d to 8 d failed to develop normally. Two representative deformed phenotypes (Figure 5A) were observed, including deformity I: four instances of molting and possessing the ability to produce offspring, with a deformed wing (I-1) or unfolded (I-2) wing, and deformity II: inability to produce offspring, with an under-developed wing (II-1), partially degenerated wing (II-2), and completely degenerated wing (II-3). Specifically, after the 2-day exposure of the first gynoparae to 0.01% kinoprene, with the exception of the blank control, gynoparae failed to develop into normal adults with intact wing and reproductive ability, and 89% and 10% of them developed into deformity I and deformity II, respectively (Figure 5B–D). With the exposure time prolonged to 4 d, 6 d, and 8 d, the proportion of nymphs that developed abnormally increased, especially nymphs suffering from deformity II (Figure 5B–D).

After the 2–8 days of the first gynoparae exposure to 0.1% kinoprene, similar phenomena were observed. To be more specific, 2-day exposure to 0.1% kinoprene resulted in the development of 33% and 67% of the gynoparae into deformity I and II, respectively (Figure 5B–D). With the exposure time prolonged to 4 d, 6 d, 8 d, the proportion of nymphs that developed abnormally increased, especially nymphs with deformity II (Figure 5B–D). Notably, even low-concentration and short-term kinoprene exposure can also influence wing differentiation. With the increasing kinoprene application time and concentration, the proportion of deformity II, with undeveloped and degenerated wings and non-reproduction, was significantly increased (Figure 5B–D), suggesting that JH signaling might be involved in regulating both wing differentiation and reproduction in gynoparae.

### 3.6. Response of JH Biosynthesis-Related Genes to Kinoprene

**Figure 6:**
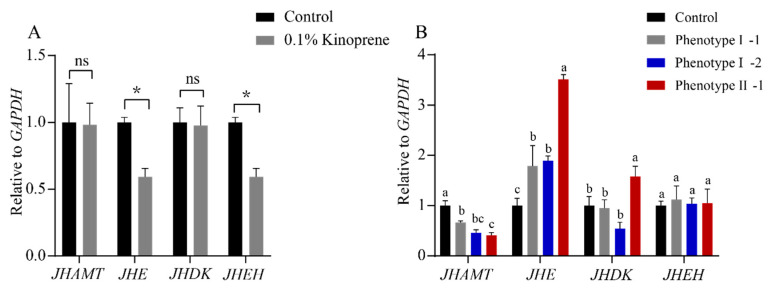
Relative expression levels of JH biosynthesis-related genes in the first instar gynoparae nymphs exposed to kinoprene for 2 days (**A**) and subsequently transferred to unsprayed leaves and collected at 6 days post-transfer (**B**). * *p* < 0.05, ns: no significant difference, a,b,c: Data are means ± SEM, different letters indicate significance at the *p* < 0.05 level as determined by one-way ANOVA following LSD test for multiple comparisons.

Some first instar gynoparae were collected after 2-day rearing on leaves sprayed with 0.1% kinoprene. The rest gynoparae were transferred to leaves with no kinoprene, collected at 6 d after transfer, and subjected to RNA extraction and quantitative validation of 4 key genes related to insect hormone biosynthesis (including JHE, JHAMT, JHDK, and JHEH). After the 2-day exposure of the first instar gynoparae nymphs to kinoprene, the expression levels of *JHE* and *JHEH* were significantly lower in treatment group than in the control, whereas the expression levels of *JHAMT* and *JHDK* were not significantly different between two groups (Figure 6A). On day 8, the expression level of *JHAMT* gene in gynoparae with deformity I and II were significantly down-regulated, compared with the control, and the expression of *JHAMT* gene in the kinoprene-treated group was gradually decreased with the increasing wing deformity degree. The expression of *JHE* gene was significantly higher in treatment group than in the control group, and it gradually increased with the increasing wing deformity degree. The expression level of *JHDK* gene in gynoparae with deformity I-1 and I-2 was not significantly different between treatment group and the control group, whereas the expression level of *JHDK* gene in gynoparae with deformity II-1 was significantly higher than that in control group. The major difference between deformity I-1 or I-2 and deformity II-1 lay in that deformity I-1 and I-2 could reproduce offspring, while deformity II-1 could not. There was no significant difference in gene expression of *JHEH* gene between the treatment group and the control group (Figure 6B).

## 4. Discussion

Gynoparae are indispensable for heteroecious holocyclic aphids to complete host transfer and reproduction mode switch. However, little is known about their morphological characteristics, development process, and embryogenesis. The present study comprehensively investigated the development dynamics (morphology, wing differentiation, and embryogenesis) of gynoparae in one holocyclic aphid species, *A. gossypii*. Our data showed that the gynoparae body was gradually enlarged with wing differentiation and deepening body color during development. The distal end of the hind leg femur and tibia joints of the adult gynoparae of the cotton aphid appeared black, which was similar to the observation of the adult gynoparae of *Aphis glycines* in one previous study [13]. Additionally, the gynoparae of *A. gossypii* exhibited irregular bands on the abdominal tergites, and the gynoparae of *A. glycines* have weak sclerotized black bands on the abdominal tergites [45]. We also observed that the fourth instar gynoparae of the cotton aphid exhibited distinct characteristics such as visible wing buds and two nested U-shaped zones containing a series of waxy secreta spots on the brownish abdomen (Figure 2A).

The formation process of embryonic sexual females was firstly revealed by observing the ovarian development of gynoparae of the cotton aphid. The fourth instar nymph stage is a critical period for ovarian development and embryonic sexual female formation. The embryos were not formed in the ovaries of the gynoparae until the fourth instar nymph stage, and red compound eyes of the embryos were also observed in the ovaries of dissected fourth instar gynoparae. Eventually, these embryos (namely embryonic sexual female) turned green when the gynoparae developed into adults, and only the first embryo in each ovary matured (Figure 2G–J), which was consistent with previous reports [34].

Our temporal transcriptome analysis revealed that the insect hormone synthesis pathways played an important role in the growth, development, and reproduction of the gynoparae. Our experiments on the exogenous addition of the JH analogue kinoprene indicated that JH signaling might be involved in regulating the wing differentiation and reproduction of gynoparae (Figure 5). This result was in line with one previous report that treating the gynoparae of *A. fabae* and *M. persicae* with kinoprene led to both wing deformity and reproduction abnormality [23]. In *G. rubens*, applying JHIII to nymphs destined to mature into long-winged adults resulted in the formation of short-winged adults [25]. Kinoprene has also been reported to regulate the growth, development, and reproduction of many other insects such as *Culex pipiens* and *Moina macrocopa* [46]. These studies collectively imply that the functions of kinoprene as a juvenile hormone analogue may be conserved across various insects. In our study, we further confirmed that the impact of kinoprene on the wing development and reproductive capacity of gynoparae was closely related to its exposure time and concentration, that even low-dose and short-term exposure to kinoprene could affect wing differentiation and formation, and that with increasing concentration and exposure time, the reproduction of gynoparae was also inhibited (Figure 5).

JH homeostasis in insects depends on its biosynthesis and degradation pathways, which are determined by four key genes. *JHAMT* is the rate-limiting enzyme gene responsible for JH synthesis, while *JHE*, *JHEH*, and *JHDK* catalyze the metabolism of juvenile hormone [47,48]. These key enzyme genes have been widely reported to contribute to the growth, development, and reproduction of insects by regulating JH homeostasis. Emerging evidence suggests that insect wing polyphenism is governed by diverse genes and regulatory networks, which depend on the taxon under study [49]. Hence, it is speculated that JH might indirectly influence wing development pathways through downstream regulatory factors. For example, silencing the *JHE* gene in *T. castaneum* leads to multiple growth abnormalities such as adult mortality, molting failure, and wing deformities [50]. The knockdown of the *JHE* gene in *Helicoverpa armigera* and *Spodoptera litura* also leads to partial larval and pupal mortality as well as deformed pupae [51,52]. In this study, the expression levels of these four key genes in gynoparae were altered after the exposure of first instar nymphs to 0.1% kinoprene, and 2-day exposure significantly down-regulated the expression levels of *JHE* and *JHEH*, but it did not affect those of *JHDK* and *JHAMT* (Figure 6A). Based on our observation that kinoprene induced wing deformity and inhibited the reproduction of gynoparae, we speculated that the reduction in JH biosynthesis might disrupt the homeostasis of juvenile hormone, thereby affecting the wing development and reproductive ability of gynoparae. After kinoprene treatment, the expression levels of *JHAMT* and JHE were significantly down-regulated and up-regulated in deformity I, respectively (Figure 6B). *JHAMT* gene has been found to be closely related to the biosynthesis of JH, and the down-regulation of *JHAMT* in *Manduca sexta* leads to an 82% reduction in the synthesis of JH in vitro [53]. Taken together, our results imply that the down- and up-regulation of *JHAMT* and *JHE* lead to the reduction in endogenous JH, further causing the wing deformity of kinoprene-treated gynoparae. Interestingly, the expression levels of *JHE* and *JHDK* genes were significantly higher in gynoparae suffering from deformity II than in gynoparae suffering from deformity I (Figure 6B), suggesting that these two genes are particularly important in the reproduction of gynoparae. JHE and JHDK are enzymes catalyzing the degradation of JH, and thus *JHE* and *JHDK* can precisely regulate JH concentrations in insects [54]. The application of the JH analogue kinoprene led to abnormal wing development and reproductive capacity of the gynoparae, which might be due to the important role of *JHE* and *JHEH* genes in the response to kinoprene. After exogenous kinoprene exposure was ceased, gynoparae may maintain the homeostasis of endogenous hormones by regulating the gene expression of *JHE*, *JHDK*, and *JHAMT* genes. However, given the gene pleiotropy, the mRNA expression changes do not always directly reflect the function of the gene [55]. In future studies, we will further determine the impact of related genes on wing formation and reproduction in gynoparae through phenotypic validation and RNA interference.

## 5. Conclusions

In this study, the morphological characteristics, developmental dynamics, and ovarian maturation process of gynoparae were investigated in the cotton aphid *A. gossypii*. Through temporal transcriptome analysis, we screened four key genes related to juvenile hormone synthesis and degradation, which might be involved in regulating wing differentiation and reproduction in gynoparae. We found that the exogenous application of kinoprene, a juvenile hormone analog, altered the expression of these key genes, thus disrupting the homeostasis of JH in gynoparae across their development, ultimately influencing the wing differentiation and reproductive ability of gynoparae.

## Data Availability

All data and materials needed to evaluate the conclusions in the paper are present in the paper and/or the Supplementary Material. Additional data related to this paper may be requested from the authors.

## Supplementary Materials

The following supporting information can be downloaded at: https://www.mdpi.com/article/10.3390/insects16060559/s1, Figure S1: Verification of RNA-Seq results of nine genes randomly selected from Cluster 1 to 12 by RT-qPCR. Table S1: Transcriptome quantitative validation primers. Table S2: The taxonomic statistics of sexupara and gynopare aphid. Table S3: Biology parameters of gynoparae across their development. Table S4: Summary of RNA-Seq data. Table S5: Gene information related to hormone synthesis pathway.

## References

[1] Mistral P., Vanlerberghe-Masutti F., Elbelt S., Boissot N. (2021). *Aphis gossypii*/*Aphis frangulae* collected worldwide: Microsatellite markers data and genetic cluster assignment. Data Brief.

[2] Elbanhawy A.A., Elsherbiny E.A., Abd El-Mageed A.E., Abdel-Fattah G.M. (2019). Potential of fungal metabolites as a biocontrol agent against cotton aphid, *Aphis gossypii* Glover and the possible mechanisms of action. Pestic. Biochem. Physiol..

[3] Heilsnis B., Mahas J.B., Conner K., Pandey S., Clark W., Koebernick J., Srinivasan R., Martin K., Jacobson A.L. (2023). Characterizing the vector competence of *Aphis gossypii*, *Myzus persicae* and *Aphis craccivora* (Hemiptera: Aphididae) to transmit cotton leafroll dwarf virus to cotton in the United States. J. Econ. Entomol..

[4] Nam H.Y., Park Y., Lee J.H. (2019). Population Genetic Structure of *Aphis gossypii* Glover (Hemiptera: Aphididae) in Korea. Insects.

[5] Peng X., Qiao X., Chen M. (2017). Responses of holocyclic and anholocyclic *Rhopalosiphum padi* populations to low-temperature and short-photoperiod induction. Ecol. Evol..

[6] Le Trionnaire G., Hardie J., Jaubert-Possamai S., Simon J., Tagu D. (2008). Shifting from clonal to sexual reproduction in aphids: Physiological and developmental aspects. Biol. Cell.

[7] Lee Y., Thieme T., Kim H. (2021). Complex evolution in *Aphis gossypii* group (Hemiptera: Aphididae), evidence of primary host shift and hybridization between sympatric species. PLoS ONE.

[8] Sandrock C., Razmjou J., Vorburger C. (2011). Climate effects on life cycle variation and population genetic architecture of the black bean aphid, *Aphis fabae*. Mol. Ecol..

[9] Emden H.F., Harrington R. (2007). Aphids as Crop Pests.

[10] Moran N.A. (1992). The evolution of aphid life cycles. Annu. Rev. Entomol..

[11] Hales D.F., Wellings P.W., Parkes R.A. (1989). Investment in gynoparae and males by *Myzus persicae* (Sulzer). Funct. Ecol..

[12] Nam K.J., Hardie J. (2014). Chemical aspects of host-acceptance behaviour in the bird cherry-oat aphid *Rhopalosiphum padi*: Host-acceptance signals used by different morphs with the same genotype. Physiol. Entomol..

[13] Tian Z.Q., Wang S.J., Bai B., Liu J., Zhao K.J. (2018). A morphological study on autumnal morphs of *Aphis glycines* (Hemiptera: Aphididae). J. Asia-Pac. Entomol..

[14] Xu K.K., Pan B.Y., Wang Y.Y., Ren Q.Q., Li C. (2020). Roles of the PTP61F Gene in Regulating Energy Metabolism of *Tribolium castaneum* (Coleoptera: Tenebrionidae). Front. Physiol..

[15] Horodyski F. (1992). Molecular Analysis of an Allatotropin in Manduca Sexta.

[16] Wu Z., Yang L., He Q., Zhou S. (2021). Regulatory Mechanisms of Vitellogenesis in Insects. Front. Cell Dev. Biol..

[17] Luo W., Liu S., Zhang W., Yang L., Huang J., Zhou S., Feng Q., Palli S.R., Wang J., Roth S. (2021). Juvenile hormone signaling promotes ovulation and maintains egg shape by inducing expression of extracellular matrix genes. Proc. Natl. Acad. Sci. USA.

[18] Leyria J., Benrabaa S., Nouzova M., Noriega F.G., Tose L.V., Fernandez-Lima F., Orchard I., Lange A.B. (2022). Crosstalk between Nutrition, Insulin, Juvenile Hormone, and Ecdysteroid Signaling in the Classical Insect Model, *Rhodnius prolixus*. Int. J. Mol. Sci..

[19] Hansen I.A., Attardo G.M., Rodriguez S.D., Drake L.L. (2014). Four-way regulation of mosquito yolk protein precursor genes by juvenile hormone-, ecdysone-, nutrient-, and insulin-like peptide signaling pathways. Front. Physiol..

[20] Roy S., Saha T.T., Zou Z., Raikhel A.S. (2018). Regulatory Pathways Controlling Female Insect Reproduction. Annu. Rev. Entomol..

[21] Smykal V., Raikhel A.S. (2015). Nutritional Control of Insect Reproduction. Curr. Opin. Insect Sci..

[22] Gao J., Guo H.J., Sun Y.C., Ge F. (2018). Juvenile hormone mediates the positive effects of nitrogen fertilization on weight and reproduction in pea aphid. Pest Manag. Sci..

[23] Hardie J., Lees A.D. (1985). The induction of normal and teratoid viviparae by a juvenile hormone and kinoprene in two species of aphids. Physiol. Entomol..

[24] Mittler T.E., Nassar S.G., Staal G.B. (1976). Wing development and parthenogenesis induced in progenies of kinoprene-treated gynoparae of *Aphis fabae* and *Myzus persicae*. J. Insect Physiol..

[25] Zera A.J., Denno R.F. (1997). Physiology and ecology of dispersal polymorphism in insects. Annu. Rev. Entomol..

[26] Dingle H., Winchell R. (1997). Juvenile hormone as a mediator of plasticity in insect life histories. Arch. Insect Biochem. Physiol..

[27] McCaffery A.R., Page W.W. (1978). Factors influencing the production of long-winged Zonocerus variegatus. J. Insect Physiol..

[28] Ishikawa A., Gotoh H., Abe T., Miura T. (2013). Juvenile hormone titer and wing-morph differentiation in the vetch aphid Megoura crassicauda. J. Insect Physiol..

[29] Niitsu S., Lobbia S., Kamito T. (2011). In vitro effects of juvenile hormone analog on wing disc morphogenesis under ecdysteroid treatment in the female-wingless bagworm moth *Eumeta variegata* (Insecta: Lepidoptera, Psychidae). Tissue Cell.

[30] Kawasaki H., Shahin R., Fujimoto S. (2023). Proliferative and preparative cell divisions in wing discs of the last larval instar are regulated by different hormones and determine the size and differentiation of the wing of Bombyx mori. J. Insect Physiol..

[31] Lin X.D., Lavine L.C. (2018). Endocrine regulation of a dispersal polymorphism in winged insects: A short review. Curr. Opin. Insect Sci..

[32] Hardie J. (1981). Juvenile hormone and photoperiodically controlled polymorphism in *Aphis fabae*: Prenatal effects on presumptive oviparae. J. Insect Physiol..

[33] Blackman R.L., Eastop V.F. (2007). Aphids on the World’s Herbaceous Plants and Shrubs.

[34] Ji J.C., Huangfu N.B., Luo J.Y., Gao X.K., Niu L., Zhang S., Cui J.J. (2021). Insights into wing dimorphism in worldwide agricultural pest and host-alternating aphid *Aphis gossypii*. J. Cotton Res..

[35] Huangfu N.B., Shi Q.Y., Chen L.L., Ma X.Y., Zhang K.X., Li D.Y., Wang L., Zhu X.Z., Ji J.C., Luo J.Y. (2022). Comparative transcriptional analysis and identification of hub genes associated with wing differentiation of male in *Aphis gossypii*. J. Cotton Res..

[36] Ji J.C., Shi Q.Y., Zhang K.X., Chen L.L., Zhu X.Z., Li D.Y., Gao X.K., Niu L., Wang L., Luo J.Y. (2023). Sexually dimorphic morphology, feeding behavior and gene expression profiles in cotton aphid *Aphis gossypii*. Pest Manag. Sci..

[37] Lü J.L., Wang L.Y., Zhang K.X., Li D.Y., Gao M.X., Guo L.X., Tang Z.J., Gao X.K., Zhu X.Z., Wang L. (2024). Morphological characteristics, developmental dynamics, and gene temporal expressions across various development stages of Aphis gossypii sexual female. J. Cotton Res..

[38] Kim D., Langmead B., Salzberg S.L. (2015). HISAT: A fast spliced aligner with low memory requirements. Nat. Methods.

[39] Zhang S., Gao X.K., Wang L., Jiang W.L., Su H.H., Jing T.X., Cui J.J., Zhang L.J., Yang Y.Z. (2022). Chromosome-level genome assemblies of two cotton-melon aphid *Aphis gossypii* biotypes unveil mechanisms of host adaption. Mol. Ecol. Resour..

[40] Pertea M., Pertea G.M., Antonescu C.M., Chang T.C., Mendell J.T., Salzberg S.L. (2015). StringTie enables improved reconstruction of a transcriptome from RNA-seq reads. Nat. Biotechnol..

[41] Eddy S.R. (1998). Profile hidden Markov models. Bioinformatics.

[42] Love M.I., Huber W., Anders S. (2014). Moderated estimation of fold change and dispersion for RNA-seq data with DESeq2. Genome Biol..

[43] Livak K.J., Schmittgen T.D. (2001). Analysis of relative gene expression data using real-time quantitative PCR and the 2^−ΔΔCT^ method. Methods.

[44] Ma K.S., Li F., Liang P.Z., Chen X.W., Liu Y., Gao X.W. (2016). Identification and Validation of Reference Genes for the Normalization of Gene Expression Data in qRT-PCR Analysis in *Aphis gossypii* (Hemiptera: Aphididae). J. Insect Sci..

[45] Kim H., Hoelmer K.A., Lee W., Kwon Y.D., Lee S. (2010). Molecular and Morphological Identification of the Soybean Aphid and Other *Aphis* Species on the Primary Host *Rhamnus davurica* in Asia. Ann. Entomol. Soc. Am..

[46] Du Y.Q., Cai J.Y., Deng X.Q., Liang W.W., Hu X.L. (2023). Growth, Reproduction, and Transgenerational Effects of Kinoprene on *Moina macrocopa*. Bull. Environ. Contam. Toxicol..

[47] Lyu Z.H., Li Z.X., Cheng J., Wang C.Y., Chen J.X., Lin T. (2019). Suppression of Gene Juvenile Hormone Diol Kinase Delays Pupation in *Heortia vitessoides* Moore. Insects.

[48] Zhang Z.J., Liu X.J., Shiotsuki T., Wang Z.S., Xu X., Huang Y.P., Li M.W., Li K., Tan A.J. (2017). Depletion of juvenile hormone esterase extends larval growth in *Bombyx mori*. Insect Biochem. Mol. Biol..

[49] Chen S.J., Zhang J.L., Ma W.J., Wu H.J., Li Y., Shen X.X., Xu H.J. (2023). FoxO and rotund form a binding complex governing wing polyphenism in planthoppers. iScience.

[50] Xu Z., Yan R., Qian J., Chen D., Guo Y., Zhu G., Wu H., Chen M. (2022). RNAi-mediated knockdown of juvenile hormone esterase causes mortality and malformation in *Tribolium castaneum*. Entomol. Res..

[51] Sarkar S., Kalia V.K. (2023). Silencing of juvenile hormone epoxide hydrolase gene in *Spodoptera litura* (Lepidoptera: Noctuidae) by oral delivery of double-stranded RNA. Biologia.

[52] Vatanparast M., Kazzazi M., Mirzaie-asl A., Hosseininaveh V. (2017). RNA interference-mediated knockdown of some genes involved in digestion and development of *Helicoverpa armigera*. Bull. Entomol. Res..

[53] Yin Y., Qiu Y.W., Huang J., Tobe S.S., Chen S.S., Kai Z.P. (2020). Enzymes in the juvenile hormone biosynthetic pathway can be potential targets for pest control. Pest Manag. Sci..

[54] Chen X.F., Gao Q., Cheng H.H., Peng F., Wang C.L., Xu B.P. (2021). Molecular cloning and expression pattern of the juvenile hormone epoxide hydrolase gene from the giant freshwater prawn *Macrobrachium rosenbergii* during larval development and the moult cycle. Aquac. Res..

[55] Li Y., Zhang J. (2025). Transcriptomic and proteomic effects of gene deletion are not evolutionarily conserved. Genome Res..

