# Supplementary material for: Reproduction and Wing Differentiation of Gynoparae Are Regulated by Juvenile Hormone Signaling in Aphis gossypii
Source: Insects. 2025 May 25;16(6):559. doi: 10.3390/insects16060559 (PMC12193124; doi:10.3390/insects16060559)
Supplement: Supplementary file 1 [file insects-16-00559-s001.zip › insects-3620454-supplementary.pdf]

Figure S1. Verification of RNA-Seq results of nine genes randomly selected from Cluster 1 to 12 by RT-qPCR

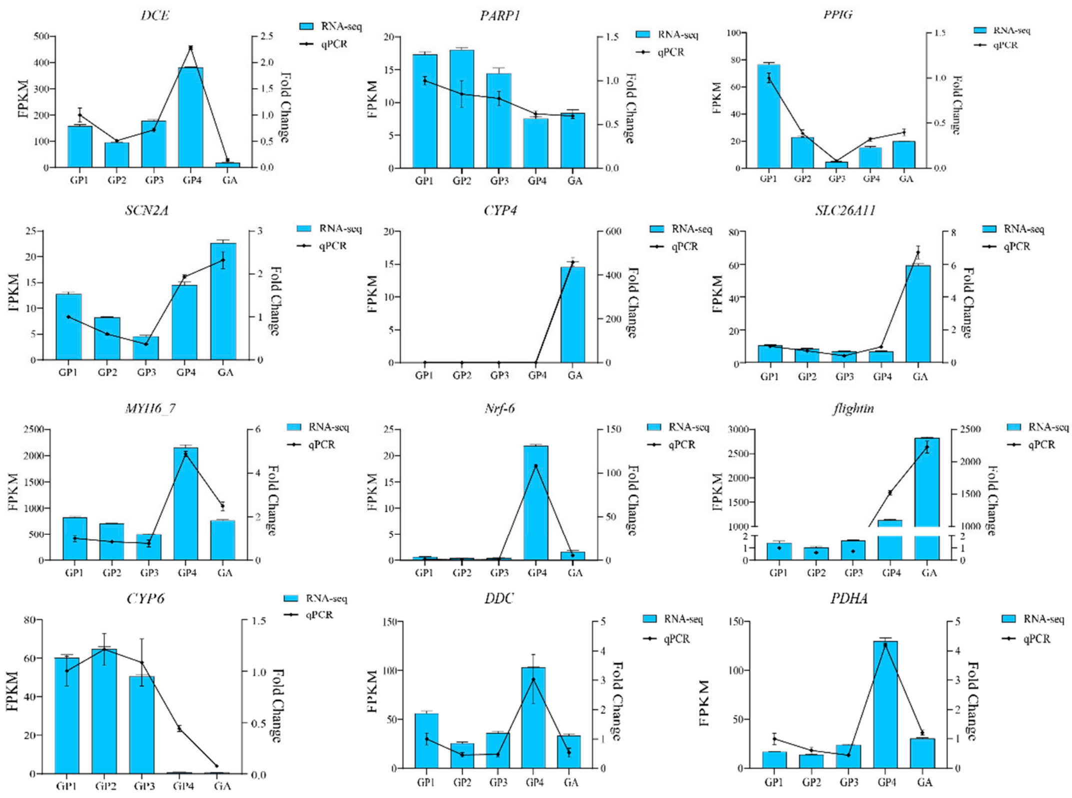

Table S1 Transcriptome quantitative validation primers

Primer Name	Primer (5'-3')
GAPDH-F	ACTACTGTTCATGCAACCACCG
GAPDH-R	GCTGCTTCCTTAACCTTATCCT
q*PARP1*-F	GCATTGGGTAACATGGTTGAGT
q*PARP1*-R	TGCCGAGAGGAACTTCAATCTT
q*PPIG*-F	TGGTCCAACTGTTCGTCCTACT
q*PPIG*-R	GGCGAGCAATCACATCATGGAA
q*CYP6*-F	TGTGCAGCCGCTCGTGAAGA

q*CYP6*-R	ACCAGCAATCCGACCGTCCA
q*SLC26A11*-F	ACATCCCGAAGACCTCACTAGC
q*SLC26A11*-R	GAGCACGCCGTATTCCAATGG
q*CYP4*-F	TCGATTGACAATGCCTGAGGTA
q*CYP4*-R	GCTGGTGGTCCTTTCAAACTTG
q*PDHA*-F	ACGGCTGGACATACTTGATGG
q*PDHA*-R	ACCACCTTTACCACGAACAACT
q*SCN2A*-F	GCTCATCTCTGTGGCGTATGCG
q*SCN2A*-R	GTCGTCTGCCGTCTGCTGTTTG
q*DCE*-F	TCTTCCACCCACTAGCCAGCAA
q*DCE*-R	AATGTGACCACCAGCACCTCTG
q*MYH6_7*-F	GATCTACAGCACGAGGCGGAAG
q*MYH6_7*-R	CGTCTGGGTGGAGGTCGAATCT
q*DDC*-F	GACACGGACAGCGAGCACAA
q*DDC*-R	CAGGGTCGCCACCACAAAGAAC
q*nrf-6*-F	GACATCTGTGTTGACCGTTTCC
q*nrf-6*-R	CCTCCGTCAGGTGTGTATTCAT
q*flightin*-F	GCACCTAAACCACCAGCCGAAT
q*flightin*-R	CCCTCTGTCGCCTGTCCATGTA

Table S2 The taxonomic statistics of sexupara and gynopare aphid.

Subfamiliy			genus			races		
	sexupara	gynopare		sexupara	gynopare		sexupara	gynopare
Eriosomatinae	128	0	Prociphilus	27	0	Pemphigini	73	0
Hormaphidinae	38	0	Pemphigus	23	0	Unknown	43	0
Adelgidae	19	1	Eriosoma	15	0	Eriosomatini	42	0
Phylloxeridae	18	0	Phylloxera	15	0	Fordini	19	0
Anoeciinae	12	0	Anoecia	12	0	Cerataphidini	16	0
Eriosomatini	6	0	Adelges	10	1	Adelgidae	10	1
Calaphidinae	2	0	Colopha	9	0	Calaphidini	2	0
Aphidinae	1	45	Pineus	9	0	Macrosiphini	1	38
Lachninae	1	0	Dysaphis	0	6	Cervaphidini	1	0
Drepanosiphinae	1	0	Aphis	0	2	Aphidini	0	7

Table S3 Biology parameters of gynoparae across the development

Parameters	1st instar nymph	2nd instar nymph	3rd instar nymph	4th instar nymph	adult
Duration period (days)	1.806 ± 0.402	1.839 ± 0.454	2.097 ± 0.396	3.645 ± 0.486	28.23 ± 2.38
Body length (mm)	0.672 ± 0.043	0.936 ± 0.069	1.181 ± 0.184	1.512 ± 0.105	1.603 ± 0.089
Body width (mm)	0.302 ± 0.026	0.431 ± 0.031	0.549 ± 0.064	0.632 ± 0.038	0.593 ± 0.048
Antenna length (mm)	0.298 ± 0.027	0.450 ± 0.031	0.623 ± 0.086	0.912 ± 0.055	0.999±0.101
Wing length (mm)					2.863±0.257

Table S4 Summary of RNA-Seq data

Sample	Total Reads	Mapped Reads	Clean reads	Clean bases	GC content	Q30(%)
GP1-1	48,714,480	46,210,321 (94.86%)	24,357,240	7,281,373,554	38.99%	90.20%
GP1-2	43,495,794	41,283,113 (94.91%)	21,747,897	6,495,004,938	37.69%	89.69%
GP1-3	43,325,640	40,810,387 (94.19%)	21,662,820	6,474,853,718	37.88%	87.02%
GP2-1	43,602,144	41,195,587 (94.48%)	21,801,072	6,516,869,382	37.12%	88.01%
GP2-2	41,279,966	39,161,138 (94.87%)	20,639,983	6,168,342,082	36.91%	89.41%
GP2-3	43,182,798	41,010,528 (94.97%)	21,591,399	6,456,698,480	36.70%	89.31%
GP3-1	42,869,844	40,688,991 (94.91%)	21,434,922	6,409,863,456	36.63%	88.12%
GP3-2	43,497,264	41,385,223 (95.14%)	21,748,632	6,502,373,836	36.65%	89.93%
GP3-3	41,108,318	39,031,371 (94.95%)	20,554,159	6,145,040,054	36.72%	88.53%
GP4-1	40,438,512	38,230,697 (94.54%)	20,219,256	6,043,080,084	36.89%	89.51%

GP4-2	42,097,528	40,172,185 (95.43%)	21,048,764	6,290,885,554	36.70%	91.70%
GP4-3	40,613,992	38,242,929 (94.16%)	20,306,996	6,069,647,226	36.84%	86.31%
GPA-1	41,123,484	38,212,200 (92.92%)	20,561,742	6,138,789,718	34.05%	85.46%
GPA-2	43,839,340	41,207,670 (94.00%)	21,919,670	6,548,394,854	33.50%	88.99%
GPA-3	40,223,526	37,733,163 (93.81%)	20,111,763	6,008,267,394	34.12%	88.24%

1

Table S5 Gene information related to hormone synthesis pathway

Sample	Gene ID	log2FC	KEGG Entry	Gene Symbol	KEGG Pathway
GP2 vs GP3	LOC114126502	3.37	K14939	CYP307A	Juvenile hormone
GP2 vs GP3	LOC114126496	2.36	K14938	NVD	Molting hormone
GP2 vs GP3	LOC114131329	2.35	K14937	CYP15A1_C1	Juvenile hormone
GP2 vs GP3	LOC114124070	1.98	K10718	JHAMT	Juvenile hormone
GP2 vs GP3	LOC114122163	-1.11	K15890	FOHSDR	Juvenile hormone
GP2 vs GP3	LOC114119988	-1.82	K15890	FOHSDR	Juvenile hormone
GP2 vs GP3	LOC114127987	-2.12	K15890	FOHSDR	Juvenile hormone
GP2 vs GP3	LOC114132522	-2.3	K01063	JHE	Juvenile hormone
GP2 vs GP3	LOC114123857	-2.37	K15890	FOHSDR	Juvenile hormone
GP3 vs GP4	LOC114132497	2.07	K14985	CYP18A1	Molting hormone
GP3 vs GP4	LOC114122163	1.83	K15890	FOHSDR	Juvenile hormone
GP3 vs GP4	LOC126549821	1.55	K10718	JHAMT	Juvenile hormone
GP3 vs GP4	LOC114129169	1.44	K10718	JHAMT	Juvenile hormone
GP3 vs GP4	LOC114132522	1.4	K01063	JHE	Juvenile hormone
GP3 vs GP4	LOC114121527	1.28	K15890	FOHSDR	Juvenile hormone
GP3 vs GP4	LOC114125573	1.19	K00128	JHE	Juvenile hormone
GP3 vs GP4	LOC114122455	1.15	K15825	JHDK	Juvenile hormone
GP3 vs GP4	LOC114125451	1.15	K01063	JHE	Juvenile hormone
GP3 vs GP4	LOC114129953	1.09	K15890	FOHSDR	Juvenile hormone
GP3 vs GP4	LOC114128007	-1.03	K10723	CYP314A1	Molting hormone

GP3 vs GP4	LOC114131898	-1.07	K10719	JHEH	Juvenile hormone
GP3 vs GP4	LOC114119318	-1.42	K15890	FOHSDR	Juvenile hormone
GP3 vs GP4	LOC114119988	-1.49	K15890	FOHSDR	Juvenile hormone
GP3 vs GP4	LOC114126496	-1.94	K14938	NVD	Molting hormone
GP3 vs GP4	LOC126548953	-2.67	K15890	FOHSDR	Juvenile hormone
GP4 vs GPA	LOC114132832	4.4	K14939	CYP308A	Molting hormone
GP4 vs GPA	LOC114132522	2.67	K01063	JHE	Juvenile hormone
GP4 vs GPA	LOC114129953	2.32	K15890	FOHSDR	Juvenile hormone
GP4 vs GPA	LOC114122185	1.61	K15890	FOHSDR	Juvenile hormone
GP4 vs GPA	LOC114127987	1.46	K15890	FOHSDR	Juvenile hormone
GP4 vs GPA	LOC114131895	1.39	K14939	CYP309A	Molting hormone
GP4 vs GPA	LOC114123857	1.29	K15890	FOHSDR	Juvenile hormone
GP4 vs GPA	LOC114122455	1.2	K15825	JHDK	Juvenile hormone
GP4 vs GPA	LOC114119318	1.12	K15890	FOHSDR	Juvenile hormone
GP4 vs GPA	LOC114132497	1.08	K14985	CYP18A2	Molting hormone
GP4 vs GPA	LOC114126502	-1.4	K14939	CYP310A	Molting hormone
GP4 vs GPA	LOC126548953	-1.44	K15890	FOHSDR	Juvenile hormone
GP4 vs GPA	LOC114121527	-1.7	K15890	FOHSDR	Juvenile hormone
GP4 vs GPA	LOC114124070	-6.43	K10718	JHAMT	Juvenile hormone
